# Supplementary material for: Chromothripsis during telomere crisis is independent of NHEJ, and consistent with a replicative origin
Source: Genome Res. 2019 May;29(5):737–49. doi: 10.1101/gr.240705.118 (PMC6499312; doi:10.1101/gr.240705.118)
Supplement: Supplemental Material [file supp_gr.240705.118_Supplemental_file_1.zip › contigs/annotated_contigs/DB56/contig.2.DB56_length_742_mean_cov_9.09703504043.docx]

**DB56_length_742_mean_cov_9.09703504043**

```
CTCAAAAAAGAAAAGGAAAAAACCCAACAGTATACTTCTCGAGGGCAGGGATTCTTTTGAAAAAAAATTCATAGATTTTATTTTTAGAA
 >chr12:99839116-99839519 - E=1e-230
CTTCTTAGACTCACAGCAAAATTGAGCAGAAATTACAAAGTTCATATATTCTCATACACAAAGCCTCCTCTACTATCAACATTCTGCTC

CAAAATGATACCTTTGTTATAATTGATGAAACTACATGGACACATCATTATCACCCAAAGTTTATAGTTTATACAGGTTCATTCTCGAT

TTATACATTCCATGAGTTGGACAAATATATAATGATGATGTATACTCACCATTATACTGTTAAATATAATAGTTTTACTGCCCTGAAAA

TCCTCAGTGCTTAACCTATTCATCCCTACCTGCCTCCAGTCCTTG|GC|GTATATATTTGAAATTATGATATCATCTTCTTGATTTTCA
                                              >chr12:99799441-99799784 - E=2e-188
CCCTTTATCATTATGTAATGATTTTTTGTCTGTTTTTATAGTTTTTTCTTGAAATCTATTTTGTCATATCAGTATAGCTACGACTGCTC

TTTTTTGATTTTTATTTGCATGGAATATCTTTGTCCATCCCCTTATTTTCAGTCTATGTGTATCTTTATAGGTGAAGTGTATTTCTTGC

AGGCAATAGATTGTTGGGTCTTTTTTTTAACCCATTCAGCTACTCTCTGTCTTTTGACTGGAGATTCTAGTCTAATTATTCAATATTAT

TGATATTAATGATAAGCACTTATCCTGCCATT
```
